# The effects of moral distress on burnout and mental well-being across healthcare and care occupations: Do age and work resources matter?

**DOI:** 10.1177/13591053251369373

**Published:** 2025-09-23

**Authors:** Tímea Zsuzsanna Popucza, Mårten Eriksson

**Affiliations:** 1 University of Gävle, Sweden

**Keywords:** moral distress, burnout, mental well-being, age, job demands-resources

## Abstract

This study investigated how moral distress contributed to burnout and mental well-being among 1318 healthcare and care professionals in Sweden using cross-sectional survey data and partial least squares structural equation modeling. Moral distress significantly increased both exhaustion and disengagement, which in turn negatively impacted mental well-being. Mediation analyses confirmed that burnout processes mediated the relationship between moral distress and mental well-being. Job control buffered the moral distress–exhaustion link, while collegial support had no effect. Linear moderation by age was non-significant, but curvilinear analysis suggested that moral distress affects burnout differently across ages. Age-group comparisons revealed that professionals under 30 and over 60 were most vulnerable to moral distress-related burnout. These findings highlight the need for preventive, age-sensitive strategies and workplace interventions that reduce moral distress and strengthen protective resources. The Swedish version of the Moral Distress Scale was validated across healthcare and care groups.

## Introduction

Moral distress arises when employees, particularly those in caring professions, encounter organizational constraints that prevent them from acting in accordance with their moral values and beliefs (Jameton, 1993). Over time, the definition has evolved, with McCarthy and Gastmans (2015) conceptualizing moral distress as “psychological, emotional, and physiological suffering” that occurs when workers face constraints that hinder morally aligned actions. This conceptualization of moral distress is valuable for everyday communication and ethical reflections on professional practice. Qualitative research supports this perspective, as individuals frequently describe their emotional responses alongside the perceived causes of their distress (Heng and Shorey, 2023; Henrich et al., 2016). In quantitative research, however, it is essential to distinguish between the causes of moral distress and its measurable effects (Oelhafen et al., 2025). As a result, measures of moral distress are often restricted to appraisal of how likely specific work characteristics give rise to such distress (Baele and Fontaine, 2021), while the consequences of moral distress are commonly measured in terms of burnout, emotional exhaustion, or turnover intentions (e.g. Fumis et al., 2017; Karakachian and Colbert, 2019; Lamiani et al., 2017; Shoorideh et al., 2015). Thus, moral distress extends beyond ethical discomfort, affecting workers’ health, well-being, and professional engagement.

Burnout was originally identified as a form of occupational stress in the caring professions characterized by exhaustion, depersonalization, and decreased professional accomplishment (Maslach and Jackson, 1981). The phenomenon has later been extended to include more professions but is still a major health problem within care, affecting both patients, organizational efficiency, and the health and well-being of personnel (Jun et al., 2021). Burnout has been defined and measured in various ways over the years, but the Oldenburg Burnout Inventory (OLBI, Halbesleben and Demerouti, 2005) is one of the most valid and reliable instruments measuring burnout (Shoman et al., 2021). The OLBI includes two dimensions, exhaustion and disengagement. Exhaustion is characterized by persistent physical, emotional, and cognitive strain, arising from prolonged exposure to demanding job conditions, ultimately draining an individual’s energy levels (Demerouti and Bakker, 2008). Disengagement reflects a process of emotional, cognitive, and behavioral withdrawal from work, often accompanied by growing negativity toward tasks, responsibilities, and the overall work environment (Demerouti and Bakker, 2008).

Although burnout is primarily linked to workplace stressors, its effects extend beyond the professional domain, impacting employees’ mental health and overall well-being (Schaufeli and Greenglass, 2001). As exhaustion and disengagement intensify, this can spill over into workers’ lives, leading to a decline in psychological well-being (Schaufeli and Greenglass, 2001). Consequently, burnout in the form of exhaustion and disengagement may serve as critical mediators in the relationship between moral distress and employees’ overall mental health.

Given the strong empirical link between moral distress and burnout, the primary aim of the present study was to investigate the relationships between moral distress and exhaustion and disengagement. This was investigated through the lens of the Job Demands-Resources (JD-R) model (Bakker et al., 2007; Demerouti et al., 2001), where moral distress, as a job demand, was hypothesized to be positively associated with exhaustion (H1a) and disengagement (H1b). Exhaustion and disengagement were further expected to be negatively related to mental well-being (H2a, H2b), positioning moral distress as an indirect predictor of reduced mental well-being (H3a), mediated through exhaustion (H3b) and disengagement (H3c).

Interestingly, a meta-analysis on aging and burnout among nurses found that older nurses reported lower levels of exhaustion and depersonalization compared to their younger counterparts (Gómez-Urquiza et al., 2017). Similarly, a Canadian study identified a linear decline in burnout risk with age among men, while in women, burnout followed a cubic trajectory, peaking among young (20–35 years) and older women (55+ years), with lower levels in midlife (Marchant et al., 2018). Consistently, data from the Swedish Social Insurance Agency suggest a curvilinear age trend in burnout diagnoses among women, peaking between ages 35 and 55 before declining, but with no second peak beyond age 55, with a similar, but less pronounced trend in men (Swedish Social Insurance Agency, 2020). These findings indicate that burnout risk varies with age, underscoring the importance of incorporating age into models examining burnout.

Lower levels of burnout and higher occupational well-being in older workers have been linked to age-related strengths in emotion regulation in older workers (see Scheibe et al., 2021 for an overview). These advantages are theoretically grounded in the motivational framework of Socioemotional Selectivity Theory (SST; Carstensen et al., 1999, 2003), suggesting that as individuals age, they prioritize emotionally meaningful goals and experiences, enhancing their motivation- and proficiency in regulating emotions. A heightened focus on emotion regulation might help older workers to cope with the emotional impact of moral distress, reducing their risk of burnout, compared to that of younger workers. Therefore, our second aim was to investigate a possibly moderating role of age on the associations between moral distress and burnout by hypothesizing that higher age reduces exhaustion (H4a) and disengagement (H4b) in response to moral distress.

A third aim was to investigate how the job resources in the form of influence at work (control) and social support from colleagues mitigate the impact of moral distress and burnout (H5a, H5b, H6a, H6b). The JD-R model (Bakker et al., 2007) posits that job resources buffer the negative impact of job demands. Control refers to the degree of autonomy employees feel they have in decision-making and task execution. Greater autonomy can reduce psychological strain by allowing employees to better manage work demands and prevent feelings of helplessness (Abdolmaleki et al., 2019; Demerouti et al., 2001). Similarly, social support from colleagues often mitigates burnout by fostering emotional validation, shared problem-solving, and collective coping strategies (Bakker et al., 2007). Social support was also inversely correlated with moral distress among nursing students (Krautscheid et al., 2020), suggesting its protective effect against moral distress. By integrating these job resources, we investigate whether control and social support from colleagues serve as protective factors that moderate the relationship between moral distress and burnout. However, while social support from a superior is often considered a strong resource in JD-R studies (Bakker et al., 2007; Demerouti et al., 2001), its role in moral distress remains unclear. It could either support the employee’s position in what the right way to act would be, or support the enforced behavior on the employee that counteracts this position and causes moral distress. Because of this ambiguous character of social support from a superior, it was not included in the model.

Finally, a fourth aim was to validate the Swedish adaptation of the Moral Distress-Appraisal Scale (Baele and Fontaine, 2021) and examine whether it functions consistently across healthcare and care professionals.

Consequently, the following hypotheses were formulated:

**H1a**: Moral distress is positively associated with exhaustion.
**H1b**: Moral distress is positively associated with disengagement.
**H2a**: Exhaustion is negatively associated with mental well-being.
**H2b**: Disengagement is negatively associated with mental well-being.
**H3a**: Moral distress has a negative indirect effect on mental well-being.
**H3b**: Exhaustion mediates the relationship between moral distress and mental well-being, such that moral distress increases exhaustion, which in turn reduces mental well-being.
**H3c**: Disengagement mediates the relationship between moral distress and mental well-being, such that moral distress increases disengagement, which in turn reduces mental well-being.
**H4a:** The positive relationship between moral distress and exhaustion is moderated by age, such that this relationship is weaker for older workers, compared to younger workers.
**H4b:** The positive relationship between moral distress and disengagement is moderated by age, such that this relationship is weaker for older, compared to younger workers.
**H5a:** The positive relationship between moral distress and exhaustion is moderated by control, such that this relationship is weaker when individuals have greater influence at work.
**H5b:** The positive relationship between moral distress and disengagement is moderated by control, such that this relationship is weaker when individuals have greater influence at work.
**H6a**: The positive relationship between moral distress and exhaustion is moderated by collegial support, such that this relationship is weaker when individuals perceive greater support from their colleagues.
**H6b**: The positive relationship between moral distress and disengagement is moderated by collegial support, such that this relationship is weaker when individuals perceive greater support from their colleagues.

**Figure 1.**
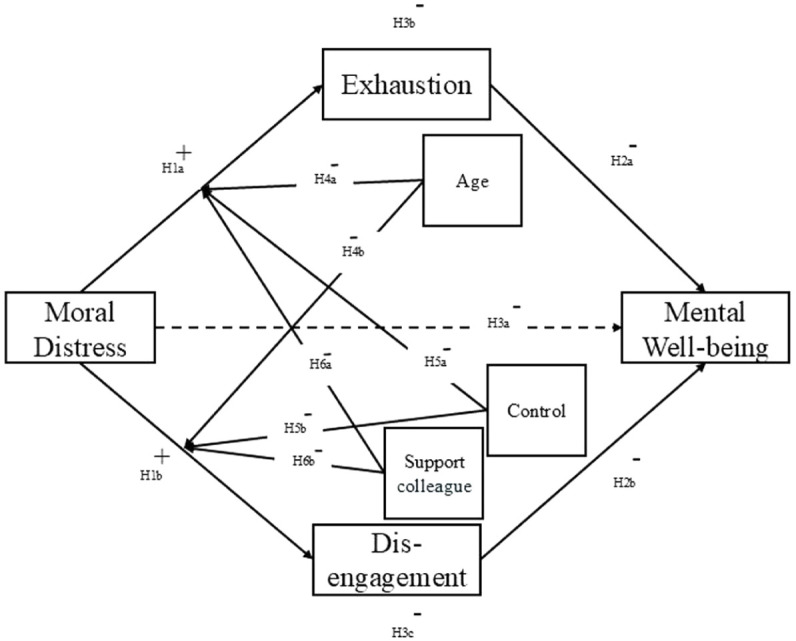
The proposed model and hypotheses.

The proposed model is presented in Figure 1.

## Methods

We employed a cross-sectional design, collecting data through an online survey. Data collection was approved in advance by *the Swedish Ethical Review Authority* (nr 2023-08117-01).

### Data collection

Data for this study were drawn from a larger dataset on work characteristics and health in an aging workforce. Participants were recruited via employer emails and social media between February 17 and June 24, 2024. Participation was voluntary and anonymous, and informed consent was obtained digitally. For this study, inclusion criteria were: (1) current employment of at least 40% full-time in (2) healthcare (e.g. nurse, doctor, physiotherapist, psychologist) or other care occupations in non-healthcare contexts (e.g. social worker, nanny, eldercare worker), and (3) regular engagement in direct care work involving interaction with patients or care recipients. Exclusion criteria included: (1) being on sick or personal leave for more than 2 weeks at the time of participation, or (2) working in non-care-related human service occupations (e.g. retail, customer service, hotel and restaurant staff, etc.).

### Measures

*Moral distress* was assessed using the Moral Distress-Appraisal Scale (MDAS; Baele and Fontaine, 2021), an eight-item, context-independent measure originally developed within healthcare. The scale includes both positive and negative items (e.g. *“I am prevented from carrying out my work in a way that I believe is morally right”* and *“I am supported to act ethically”*), rated on a 6-point Likert scale. The scale was translated into Swedish by the authors and back-translated by a native English speaker, with refinements made through group discussions.

*Burnout* was measured using the Swedish version of the Oldenburg Burnout Inventory (OLBI; Peterson et al., 2008), which assesses exhaustion and disengagement across 16 items (8 per dimension). Exhaustion captures extreme fatigue (e.g. *“There are days when I feel tired before I arrive at work.”*), while disengagement reflects detachment from work (e.g. *“Lately, I tend to think less at work and do my job almost mechanically.”*). Items were rated on a 4-point Likert scale.

*Mental well-being* was assessed using the Swedish version of the short Warwick-Edinburgh Mental Well-Being Scale (SWEMWBS; Ringdal et al., 2018; Tennant et al., 2007), which includes seven positively worded items related to positive affect, relationships, and functioning (e.g. *“I’ve been feeling optimistic about the future.”*). Responses were rated on a 5-point Likert scale.

*Work resources* were measured using the *Control* and *Support from Colleagues* dimensions from the Copenhagen Psychosocial Questionnaire III (COPSOQ III; Berthelsen et al., 2020; Burr et al., 2019). Influence at work (four items) assesses employees’ control over tasks and decisions (e.g. *“Do you have any influence on what you do at work?”*). Social Support from Colleagues was assessed by two items (e.g. *“Your colleagues listen to your work-related problems”*). Both resources were rated on a 5-point Likert scale.

### Participants

A priori power analysis using G*Power (Faul et al., 2009) indicated a required sample size of 1134 (small effect), 162 (medium effect), and 77 (large effect) at 0.80 power. The final sample included 1318 participants: 865 (65.6%) healthcare professionals and 453 (34.4%) in other care-related roles (e.g. social worker, counselor, preschool teacher, nanny, elderly care, etc.). The majority were women (1241 participants, 94.2%). Ages ranged from 22 to 72 years (*M* = 46.2, SD = 10.4), distributed as follows: 68 (5.2%) under 30; 332 (25.2%) aged 30–39; 356 (27.0%) aged 40–49; 416 (31.6%) aged 50–59; 146 (11.1%) aged 60 or older. Participant characteristics are detailed in Table 1. Missing data (*n* = 16) were addressed using mean replacement to retain cases in analyses.

**Table 1.** Participant characteristics.

Category	n	%
Gender		
Female	1241	94.2
Male	70	5.3
Unspecified	7	0.5
Age distribution		
Mean age	46.2	
Standard deviation	10.4	
22–29 years	68	5.2
30–39 years	332	25.2
40–49 years	356	27.0
50–59 years	416	31.6
60+ years	146	11.1
Profession		
Healthcare	865	65.6
Care	453	34.4
Education		
Elementary school or less	10	0.8
High school	126	9.6
<3 years post-secondary	167	12.7
3 years post-secondary	251	19.0
>3 years post-secondary	764	58.0
Experience in current role		
<3 months	13	1.0
3–12 months	58	4.4
1–3 years	172	13.1
3–5 years	144	10.9
5–10 years	256	19.4
>10 years	675	51.2

### Analysis

We used Partial Least Squares Structural Equation Modeling (PLS-SEM) to validate the measurement model and test hypotheses. Based on theory and measurement rationale (Hair et al., 2022), moral distress was modeled as a formative construct, while control, support, burnout, and mental well-being were treated as reflective constructs. For the formative construct, we assessed dimensionality (PCA), collinearity (VIF), and indicator relevance (weights and loadings). For reflective constructs, we evaluated dimensionality, reliability (Cronbach’s alpha, composite reliability, outer loadings), and validity (convergent: AVE; discriminant: HTMT). The structural model was assessed using VIF for collinearity, bootstrapping for path coefficient significance, and standardized beta coefficients. Explanatory power and predictive relevance were evaluated via *R²*, *f²*, and *Q²*. Multigroup analyses (cB-MGA) compared path coefficients and effect sizes across age groups. MICOM tested construct validity and model reliability across professional groups (Hair et al., 2022).

## Results

### Measurement model evaluation

PCA assessed the dimensionality of all constructs. Except for disengagement, constructs were unidimensional (eigenvalues >1, variance explained >50%). Kaiser-Meyer-Olkin (KMO) values (0.614–0.977) confirmed data suitability for PCA. For disengagement, two components had eigenvalues >1 (3.39, 1.06), explaining 55.8% of variance (42.5%, 13.3%), indicating borderline multidimensionality, with factors aligning by item phrasing, suggesting a wording effect. Given the borderline eigenvalues, theoretical grounding, and prior research supporting unidimensionality, disengagement was retained as a single construct.

The evaluation of the formative Moral Distress construct revealed collinearity concerns based on VIF values. Indicator 8 had a VIF value of 5.014, slightly above the recommended threshold of 5.00 (Hair et al., 2022). Other indicators ranged from 3.246 to 4.619, indicating moderate collinearity. Removing Indicator 8 (*“I am helped to work in a way that I believe is morally right”*) reduced all VIF values (1.885–4.162), mitigating multicollinearity. Four indicators (MD2, MD5, MD6, MD7) had significant outer weights (*p* < 0.05), while MD1, MD3, and MD4 were non-significant, suggesting weaker contributions. However, all indicators had significant outer loadings above 0.7 (*p* < 0.05), confirming absolute relevance. This supports the validity of the formative Moral Distress construct (Supplemental Materials Table S1).

To evaluate the reflective constructs (exhaustion, disengagement, mental well-being, influence at work, and collegial support), we first examined outer loadings. Most indicators showed strong reliability (⩾0.7), except for disengagement item 7 (*“I find my work to be a positive challenge”*, loading = 0.298), which was removed. Item 13 (*“This is the only type of work I can imagine doing”*, loading = 0.524) was also removed due to initial concerns about convergent validity. Convergent validity was assessed using average variance extracted (AVE), with values > 0.50 indicating that constructs explain at least 50% of indicator variance (Hair et al., 2022). Initially, disengagement fell below this threshold but surpassed it after item 13 was removed. Reliability was evaluated using Cronbach’s alpha, reliability coefficient (*ρa*), and composite reliability (*ρc*), with all constructs exceeding 0.70, confirming strong internal consistency. Finally, discriminant validity was assessed via the heterotrait-monotrait ratio (HTMT), using the 0.90 threshold (Hair et al., 2022). All values fell below this limit, confirming construct distinctiveness (Supplemental Materials Table S2–S3).

### Structural model evaluation

**Figure 2.**
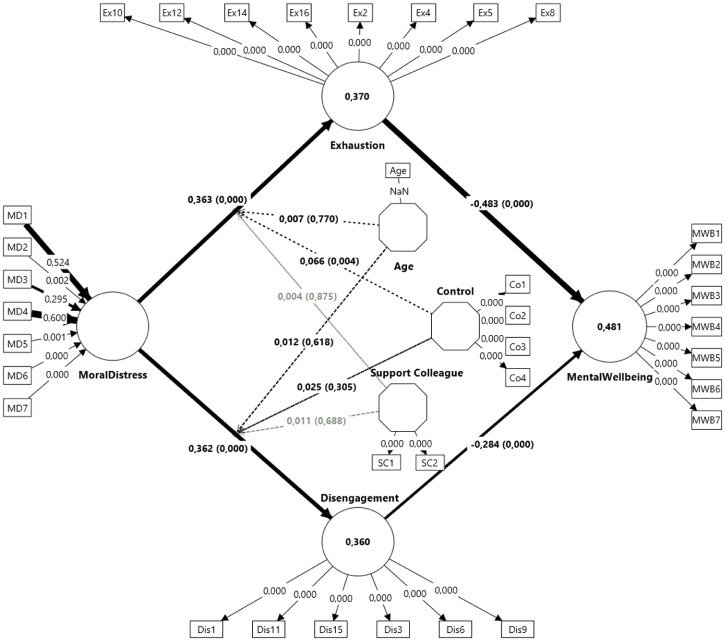
The structural model.

Structural model collinearity VIF ranged from 1.178 to 1.581, indicating no multicollinearity. Predictive power (*R*²) indicated that predictors explained 36.0% of Disengagement (*R*² = 0.360, *p* < 0.001), 37.0% of Exhaustion (*R*² = 0.370, *p* < 0.001), and 48.1% of Mental Well-being (*R*² = 0.481, *p* < 0.001) of the variance, suggesting moderate to substantial explanatory power (Hair et al., 2022; Figure 2).

Effect sizes (*f*²; Table 2) indicated that Moral Distress large effect on Disengagement (*f*² = 0.148, *p* < 0.001) and Exhaustion (*f*² = 0.151, *p* < 0.001). Exhaustion had a moderate effect (*f*² = 0.284, *p* < 0.001), while Disengagement had a small effect (*f*² = 0.098, *p* < 0.001) on Mental Well-being. Effect sizes for age on Disengagement and Exhaustion were non-significant. Interaction effects of age were negligible (*f*² ⩽ 0.002, *p* > 0.05), as were those of Control and Collegial Support (*f*² ⩽ 0.01, *p* > 0.05), indicating no moderation by age, influence at work, or Collegial Support.

**Table 2.** Path coefficients, effect sizes (f²), and confidence intervals.

Path	*β*	*p*-Value	95% CI [Lower, Upper]	*f*²	Hypothesis
Hypothesized direct effects					
Moral distress→Exhaustion	0.363	**0.000**	[0.313, 0.417]	0.151*	H1a Confirmed
Moral distress→Disengagement	0.362	**0.000**	[0.310, 0.419]	0.148*	H1b Confirmed
Exhaustion→Mental well-being	−0.483	**0.000**	[−0.530, −0.437]	0.284*	H2a Confirmed
Disengagement→Mental well-being	−0.284	**0.000**	[−0.336, −0.235]	0.098*	H2b Confirmed
Hypothesized interaction effects (moderation)					
Age×Moral distress→Exhaustion	0.007	0.770	[−0.037, 0.051]	0.000	H4a Not confirmed
Age×Moral distress→Disengagement	0.012	0.618	[−0.035, 0.060]	0.000	H4b Not confirmed
Control×Moral distress→Exhaustion	0.066	0.004	[0.022, 0.111]	0.007	H5a Not confirmed
Control×Moral distress→Disengagement	0.025	0.305	[−0.023, 0.071]	0.001	H5b Not confirmed
Collegial Support×Moral distress→Exhaustion	0.004	0.875	[−0.044, 0.052]	0.000	H6a Not confirmed
Collegial Support×Moral distress→Disengagement	0.011	0.688	[−0.041, 0.066]	0.000	H6b Not confirmed
Hypothesized indirect effects (mediation)					
Moral distress→Mental well-being (Total Indirect)	−0.278	**0.000**	[−0.318, −0.240]		H3a Confirmed
Moral distress→Exhaustion→Mental well-being	−0.175	**0.000**	[−0.210, −0.146]		H3b Confirmed
Moral Distress→Disengagement→ Mental well-being	−0.103	**0.000**	[−0.130, −0.080]		H3c Confirmed
Additional direct effects					
Age→Exhaustion	−0.075	**0.001**	[−0.118, −0.032]	0.009	
Age→Disengagement	−0.089	**0.000**	[−0.136, −0.044]	0.012	
Control→Exhaustion	−0.246	**0.000**	[−0.298, −0.192]	0.074	
Control→Disengagement	−0.222	**0.000**	[−0.275, −0.167]	0.059	
Collegial support→Exhaustion	−0.160	**0.000**	[−0.208, −0.110]	0.034*	
Collegial support→Disengagement	−0.177	**0.000**	[−0.227, −0.129]	0.041*	
Quadratic age moderation					
Z_Age²→Exhaustion	0.001	0.979	[−0.040, 0.041]	0.00	
Z_Age²→Disengagement	0.08	**0.001**	[0.033, 0.125]	0.01	
Z_Age²×Moral Distress→Exhaustion	0.061	**0.005**	[0.018, 0.102]	0.005	
Z_Age²×Moral Distress→Disengagement	0.069	**0.009**	[0.017, 0.121]	0.007	

Note. β: standardized path coefficient. Significant *p*-values (*p* < 0.05) are bold. Significant effect sizes are marked with *.

Predictive relevance (*Q*²) for Disengagement (*Q*² = 0.549), Mental Well-being (*Q*² = 0.544), Exhaustion (*Q*² = 0.516), and the overall model *Q*² (0.535) indicate predictive capacity (*p* < 0.001, Hair et al., 2022).

#### Hypothesis testing

##### Direct effects

Moral Distress exhibited positive effects on both Exhaustion (*β* = 0.363, *p* < 0.001) and Disengagement (*β* = 0.362, *p* < 0.001), confirming H1a and H1b. This suggests that higher levels of moral distress contribute to increased exhaustion and disengagement. In turn, both Exhaustion (*β* = −0.483, *p* < 0.001) and Disengagement (*β =* −0.284, *p* < 0.001) had a negative effect on Mental Well-being, confirming H2a and H2b. These results indicate that both exhaustion and disengagement have a moderate to strong detrimental effect on mental well-being (Table 2).

##### Mediation

A significant negative indirect effect of Moral Distress on Mental Well-being was found (*β* = −0.278, *p* < 0.001), confirming H3a. This indicates that moral distress is associated with lower mental well-being through its effects on exhaustion and disengagement. Specifically, the results confirmed that Moral Distress lowers Mental Well-being through Exhaustion (H3b; *β* = −0.175, *p* < 0.001) and Disengagement (H3c; *β* = −0.103, *p* < 0.001; Table 2).

##### Moderation

While Age had significant negative direct effects on Disengagement (*β* = −0.089, *p* < 0.001) and Exhaustion (*β* = −0.075, *p* = 0.001), Age neither moderated the relationship between Moral Distress and Exhaustion (*β* = 0.007, *p* = 0.770), nor the relationship between Moral Distress and Disengagement (*β* = 0.012, *p* = 0.618), indicating that the impact of moral distress on exhaustion and disengagement did not decline for older workers. Hence, H4a or H4b received no support. The work resources Control and Collegial Support were significantly associated with lower Disengagement (*β* = −0.222, *β* = −0.177,) and lower Exhaustion (*β* = −0.246, *β* = −0.160), respectively, all *p* < 0.001, suggesting that both Control and Collegial support have a protective effect on burnout. However, only Control moderated the relationship between Moral Distress and Exhaustion (*β* = 0.066, *p* = 0.004), confirming H5a. H5b was not supported. This suggests that having greater influence at work buffers the impact of moral distress on exhaustion, but it does not alter the effect on disengagement. Finally, neither H6a nor H6b were supported. Collegial Support did not moderate the effects of Moral Distress on neither Exhaustion nor on Disengagement. Hence, perceived collegial support did not weaken the impact of moral distress on burnout (Table 2).

##### Exploratory age analyses

To explore nonlinear age effects, we tested quadratic moderation (ZAge²) in the model (Table 2). ZAge² significantly predicted Disengagement (*β* = 0.08, *p* = 0.001) but not Exhaustion (*β* = 0.001, *p* = 0.979), indicating a curvilinear trend for Disengagement but not Exhaustion. ZAge² also significantly moderated the relationships between Moral Distress and both Disengagement (*β* = 0.069, *p* = 0.009) and Exhaustion (*β* = 0.061, *p* = 0.005), suggesting that the effect of Moral Distress on Burnout varies across ages. However, the small and non-significant effect sizes along with the weak beta coefficients indicated that ZAge² does not meaningfully contribute to burnout variance, neither directly nor as a moderator.

To investigate more thoroughly the moral distress–burnout relationship across age groups, we conducted a multi-group analysis without age as a moderator, comparing five groups: under 30 (*n* = 68), 30–39 (*n* = 332), 40–49 (*n* = 356), 50–59 (*n* = 416), and 60+ (*n* = 146). Path and effect size comparisons are available in Supplemental Materials, Tables S4–S6.

**Figure 3.**
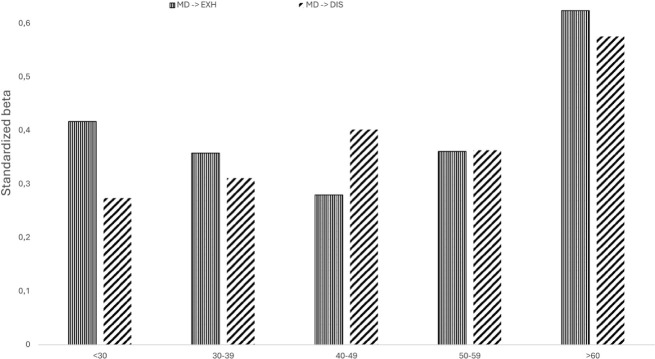
Moral distress and burnout across age groups.

The effect of Moral Distress on Exhaustion was significantly stronger for the 60+ group, compared to the under 30 group (*Δβ* = −0.208; *p* < 0.001), the 30–39 group (*Δβ* = −0.266; *p* < 0.001), the 40–49 group (*Δβ* = −0.344; *p* < 0.001) and the 50–59 group (*Δβ* = −0.263; *p* < 0.001). Similarly, the effect of Moral Distress on Disengagement was significantly stronger for the 60+ group, compared to the under 30 group (*Δβ* = −0.302; *p* < 0.001) 30–39 group (*Δβ* = −0.265; *p* < 0.001), the 40–49 group (*Δβ* = −0.174; *p* < 0.001), and the 50–59 group (*Δβ* = −0.214; *p* < 0.001). All these effect size comparisons were also significantly different. This suggests that the oldest workers are the most vulnerable to the negative impact of moral distress on both burnout dimensions. Additionally, the effect of Moral Distress on Exhaustion was significantly stronger for the under 30 group, compared to the under 30–39 group (*Δβ* = 0059; *p* < 0.001), the 40–49 group (*Δβ* = 0.137; *p* < 0.001), and the 50–59 group (*Δβ* = 0055; *p* < 0.001). However, the effect of Moral Distress on Disengagement was significantly weaker for the under 30 group, compared to the under 30–39 group (*Δβ* = −0,037; *p* < 0.001), the 40–49 group (*Δβ* = −0.128; *p* < 0.001), and the 50–59 group (*Δβ* = −0,088; *p* < 0.001). All these effect size comparisons were also significantly different. This suggests that while the youngest workers (under 30) report a higher level of exhaustion in relation to moral distress, moral distress impacts their levels of disengagement less negatively compared to middle-aged groups. Figure 3 presents paths between moral distress and burnout across age groups.

##### Professional groups

To assess the applicability of the moral distress scale among healthcare and care professionals, we evaluated construct validity and reliability across groups. Configural invariance was confirmed, indicating a consistent model structure across professional groups. MICOM results (Table 3) confirmed compositional invariances for all constructs (*ρ* ≈ 1, *p* > 0.05), supporting comparability. However, Step 3a (Equality of Means) showed significant differences for disengagement (*ΔM* = −0.201, *p* < 0.001), exhaustion (*ΔM* = −0.236, *p* = 0.001), and mental well-being (*ΔM* = 0.172, *p* = 0.001), suggesting differences in perception. Step 3b (Equality of Variances) was supported (*p* > 0.05), indicating similar response variability. These findings confirm that the moral distress scale functions consistently across professional groups; however, differences in disengagement, exhaustion, and well-being suggest that these constructs are experienced differently between groups.

**Table 3.** Compositional invariance and equality of means/variances (Step 2 & Step 3).

	Step 2: Compositional invariance		Step 3a: Equality of means		Step 3b: Equality of variances	
	*ρ*	Permutation *p*	Δ*M*	Permutation *p*	Δ*σ*²	Permutation *p*
Disengagement	1.000	0.894	**−0.201**	**0.000**	−0.037	0.613
Exhaustion	1.000	0.575	**−0.236**	**0.001**	0.037	0.632
Mental well-being	1.000	0.579	**0.172**	**0.001**	−0.132	0.183
Moral distress	0.998	0.759	−0.099	0.124	−0.109	0.159

*Note*. *ρ*: compositional invariance; Δ*M*: mean difference; Δσ²: variance difference. Compositional invariance is confirmed when ρ ≈ 1 and *p* > 0.05. Equality of means and variances is supported when Δ*M* and Δσ² have *p* > 0.05 (bolded) (Hair et al., 2024).

## Discussion

We first aimed to investigate the relationship between moral distress and burnout (exhaustion and disengagement) and its impact on workers’ mental well-being. Second, we examined whether these relationships were influenced by linear age. Third, we assessed the moderating role of job resources (influence at work and collegial support) on the moral distress-burnout link. In subsequent analyses, we explored curvilinear age effects and compared five age groups to map potential age-related differences. Fourth, we evaluated the adaptation of the Moral Distress-Appraisal Scale into Swedish and its applicability across healthcare and care professionals. The following sections discuss direct and indirect effects, moderation by job resources, age-related findings, model applicability and generalizability, as well as implications, limitations, and future directions.

### Direct effects

Our findings confirmed that moral distress significantly increases exhaustion and disengagement. This link between moral distress and burnout is well-documented, particularly in healthcare, where ethical dilemmas found to be associated with burnout (see Karakachian and Colbert, 2019; Lamiani et al., 2017 for reviews) However, while the studies included in these reviews focused on healthcare professionals, our findings highlight that moral distress is an occupational health hazard within other care professionals in Sweden as well.

Both exhaustion and disengagement strongly predicted mental well-being (Demerouti and Bakker, 2008; Halbesleben and Demerouti, 2005). Hence, burnout not only negatively impacts work-related outcomes but also affects workers’ mental health. This is consistent with findings by Maddock (2024), who identified burnout as a significant predictor of anxiety, depression, and diminished well-being among social workers. Similarly, Mbanga et al. (2019) found that burnout was a strong predictor of depression among nurses. Our findings support these studies, suggesting that both burnout dimensions negatively affect workers’ overall mental well-being.

### Mediation effect

The indirect effect of moral distress on mental well-being through exhaustion and disengagement also suggests that work-related ethical strain extends beyond the professional domain, affecting overall mental well-being. Rather than testing for traditional mediation, we excluded a direct path from moral distress to mental well-being, as both theoretical models of work stress (e.g. Job Demands-Resources,Bakker et al., 2007, and Job Demands-Control, Karasek, 1979) and previous research on moral distress have primarily focused on its impact on occupational outcomes such as burnout, job performance, and psychological strain (see Karakachian and Colbert, 2019; Lamiani et al., 2017; Roczniewska et al., 2022 for reviews). Therefore, exhaustion and disengagement were the most relevant direct outcomes and mediating pathways. However, our findings reinforce the critical role of burnout dimensions in shaping mental well-being, emphasizing the need for workplace interventions that mitigate the antecedents of exhaustion and disengagement to prevent their long-term psychological consequences.

### Job resources

Our results provide insights into the role of Control and Collegial support in shaping the effects of moral distress on exhaustion and disengagement. While both resources showed significant direct effects on burnout, their moderating effects were weak or non-significant, raising important questions about their potential as buffers against moral distress.

High Control was associated with lower exhaustion and disengagement, supporting the idea that autonomy reduces burnout by enabling employees to manage work demands in ways that align with their needs and preventing feelings of helplessness (Demerouti et al., 2001). High workplace autonomy in Abdolmaleki et al. (2019) was also negatively correlated with moral distress and burnout, suggesting that Control may protect against the negative impacts of ethical conflicts. However, our results showed that Control only moderated the relationship between moral distress and exhaustion, but not that between moral distress and disengagement. This indicates that while more control over work might help employees to actively reduce the negative effects of moral distress, it does not prevent emotional, cognitive, and behavioral withdrawal from work. Ethical dilemmas and value conflicts seem to lead to disengagement regardless of influence at work, as having control over one’s work does not resolve or decrease ethical conflicts.

Collegial support strongly reduced exhaustion and disengagement, reinforcing the protective role of workplace relationships (Bakker and Demerouti, 2007; Demerouti et al., 2001). However, it did not moderate the effects of moral distress on burnout, suggesting that while general workplace support is beneficial, it does not buffer distress from ethical dilemmas. Unlike other job demands that coworkers can help alleviate, moral distress likely requires organizational-level solutions.

### The effects of age

Although older employees reported lower levels of exhaustion and disengagement, linear age did not moderate the relationship between moral distress and burnout. This, on the one hand, suggests potential age-related advantages, making older workers less prone to burnout, potentially due to older age-related strengths in emotional regulation (SST; Carstensen et al., 2003; Scheibe et al., 2021). However, since the impact of moral distress on burnout did not decrease with age, the potential age-related strengths in emotional regulation may not be effective in mitigating these effects. This might indicate that, in the face of moral distress, emotion regulation alone is insufficient, and other solutions are needed to address the effects of moral distress.

Interestingly, further analyses revealed a nonlinear age pattern on the effect of moral distress on burnout, with the most pronounced negative impact of moral distress on burnout in the oldest workers (60+). In this group, moral distress had a significantly stronger impact on both exhaustion and disengagement, compared to all the younger groups, with the significant effect sizes further indicating meaningful differences. This suggests that as workers approach seniority, particularly after the age of 60, they become increasingly vulnerable to the adverse effects of moral distress. A possible explanation for this could be the accumulated exposure to workplace ethical dilemmas throughout a career, which may lead to a cumulative burden, making older employees more susceptible to exhaustion and disengagement when faced with moral distress (Nilsson, 2024). Additionally, age-related changes in cognitive flexibility, stress tolerance, and physiological resilience may contribute to heightened vulnerability to workplace emotional stressors, as suggested by the Strength and Vulnerability Integration (SAVI) model (Charles, 2010). Given that age had a linear negative effect on burnout, this heightened vulnerability in the 60+ group suggests that the oldest workers have a distinctly stronger susceptibility to moral distress-driven burnout. However, these findings should be interpreted with caution, as the smaller sample sizes in the 60+ (*n* = 146) group may impact the stability of the estimates.

Moreover, even among the youngest workers, moral distress had a distinct impact on burnout. While younger workers were significantly more vulnerable to exhaustion, their levels of disengagement were significantly lower in relation to moral distress compared to middle-aged groups. This suggests that the effect of moral distress manifests differently in this age group, primarily contributing to exhaustion rather than disengagement. Younger workers appear to experience heightened strain when encountering moral distress, yet they remain more engaged in their work compared to their middle-aged counterparts. Although research on how moral distress affects younger workers is scarce, this pattern may reflect differences in coping mechanisms, professional experience, or resilience levels (Singh et al., 2020). However, these findings should also be interpreted with caution, given the relatively small number of participants under 30 (*n* = 68).

The observed pattern, combined with the significant effect sizes, suggests meaningful age-related differences in the experience of moral distress, highlighting the need for further research to better understand these dynamics, and uncover the underlying mechanisms driving these age-related effects.

### Implications

The model’s explanatory and predictive power confirmed that moral distress is a robust predictor of burnout and mental well-being, supporting existing frameworks (e.g. JD-R, Bakker and Demerouti, 2007) on how workplace stressors contribute to exhaustion and disengagement, and in turn affect workers’ overall mental well-being. Notably, exhaustion had a stronger impact on mental well-being than disengagement, suggesting that burnout primarily affects well-being through physical and emotional depletion, rather than psychological withdrawal. This underscores the importance of targeting exhaustion to mitigate its effects on workers’ mental health.

The findings also indicate that moral distress requires targeted organizational interventions rather than relying solely on individual resilience or general workplace resources. While job resources such as Control and Collegial support reduce exhaustion and disengagement in general, they appear insufficient in buffering moral distress. Organizations with recurrent issues of moral distress may implement ethical support systems, clear ethical guidelines, and structured decision-making frameworks to help employees navigate moral dilemmas effectively.

Contrary to our hypotheses, older workers (60+) were the most vulnerable to the negative effects of moral distress on both burnout dimensions. Additionally, younger workers (under 30) were also more susceptible to exhaustion related to moral distress. These findings highlight the need for targeted interventions to support both early- and late-career professionals in navigating ethical challenges at work. Early career interventions might also prevent the long-term accumulation of moral distress, promoting sustainable work practices for all age groups.

The measurement model evaluation confirmed that the Swedish version of the Moral Distress-Appraisal Scale performed well, with minor adjustments improving construct validity. Invariance testing further demonstrated that the model remains consistent across both healthcare and care professionals, supporting the generalizability of moral distress as a construct in Swedish healthcare and care settings. Additionally, our findings suggest that the mechanisms linking moral distress to burnout are similar across these professions, reinforcing the model’s theoretical and practical relevance. However, observed differences in disengagement, exhaustion, and well-being indicate that while moral distress is a shared experience, likely arising from common ethical challenges and structural constraints across care-related roles, its impact may vary depending on other occupation-specific factors.

### Limitations

This study has several limitations. The cross-sectional design captures perceptions at a single time point, limiting causal interpretations, though it still provides valuable insights into associations. Self-reported one-time measures for both independent and dependent variables increase the risk of common method bias (CMB; Podsakoff et al., 2003). However, to mitigate this risk, participants were assured anonymity, response formats were varied, and a collinearity test was conducted. VIF values remained below conventional thresholds (Hair et al., 2022; Kock, 2015), suggesting that CMB was unlikely to have influenced results. Sample composition also presents limitations, as employer-based recruitment had low response rates, leading to the use of social media, which may have introduced self-selection bias. Most participants (58%) had more than 3 years of post-secondary education. This may reflect better coping strategies, wellbeing, and access to mental health resources, which might reduce distress among most of them. The findings may not fully represent less-educated populations, who might experience burnout differently. The sample skewed toward experienced professionals (51% with >10 years), raising the possibility of survivorship bias, where those most affected by burnout may have already left the profession. This may lead to underestimation of distress, especially among early-career professionals. The predominantly female sample (94.2%) aligns with gender distributions in healthcare and care professions (National Board of Health and Welfare, 2023) but limited gender comparisons. Regarding the measurements, the Moral Distress Scale revealed some collinearity concerns, however, the removal of one indicator improved collinearity. Finally, moral distress was the only work demand examined, limiting insight into how it interacts with other job demands in predicting burnout and mental well-being.

### Future directions

Considering our limitations, future longitudinal and experimental studies might clarify causal relationships between moral distress, burnout, and well-being. Expanding the scope to include additional work demands and job resources would provide a more comprehensive understanding of workplace stressors and protective factors. For example, research could explore resources (e.g. psychological safety climate, individual coping strategies) that can mitigate the effect of moral distress, and design interventions for professionals facing ethical challenges. Increasing sample diversity (e.g. gender and ethnicity) could also improve the generalizability of findings.

Future research could also focus on healthcare professionals at both ends of the career span examining how the youngest and oldest professionals experience and manage moral distress, including workplace dynamics and self-regulatory processes. While older workers generally report lower burnout, moral distress appears to have a stronger impact on their exhaustion and disengagement, making them particularly vulnerable in ethically challenging environments. Similarly, at an early career stage, professionals can be especially vulnerable to the effect of moral distress, manifesting in higher exhaustion. Further evidence of these effects is essential for developing targeted strategies to support novice and retain experienced healthcare professionals.

## Conclusion

Our findings confirmed that moral distress increases exhaustion and disengagement, and both burnout dimensions negatively impacted mental well-being, reinforcing the importance of work-related factors for overall psychological health. Mediation analyses also showed that moral distress indirectly undermines mental well-being through burnout, emphasizing exhaustion and disengagement as key pathways.

Regarding moderation, only Control buffered the moral distress–exhaustion link, while Collegial support had no effect. Age did not linearly moderate the moral distress-burnout relationship, suggesting older workers are not inherently more resilient. A subsequent curvilinear moderation analysis indicated that moral distress affects burnout differently across ages. Age-group comparisons revealed that the youngest (<30) and oldest (60+) workers were most vulnerable to burnout when experiencing moral distress.

These findings highlight the need for preventive strategies across age groups, particularly for workers under 30 and over 60. Addressing moral distress and enhancing workplace resources, especially job Control, may be essential for promoting psychological health among healthcare and care professionals.

## Supplemental Material

sj-docx-1-hpq-10.1177_13591053251369373 – Supplemental material for The effects of moral distress on burnout and mental well-being across healthcare and care occupations: Do age and work resources matter? Supplemental material, sj-docx-1-hpq-10.1177_13591053251369373 for The effects of moral distress on burnout and mental well-being across healthcare and care occupations: Do age and work resources matter? by Tímea Zsuzsanna Popucza and Mårten Eriksson in Journal of Health Psychology

sj-xlsx-2-hpq-10.1177_13591053251369373 – Supplemental material for The effects of moral distress on burnout and mental well-being across healthcare and care occupations: Do age and work resources matter? Supplemental material, sj-xlsx-2-hpq-10.1177_13591053251369373 for The effects of moral distress on burnout and mental well-being across healthcare and care occupations: Do age and work resources matter? by Tímea Zsuzsanna Popucza and Mårten Eriksson in Journal of Health Psychology
