# Supplementary material for: The effects of moral distress on burnout and mental well-being across healthcare and care occupations: Do age and work resources matter?
Source: J Health Psychol. 2025 Sep 23;31(6):2399–415. doi: 10.1177/13591053251369373 (PMC13070131; doi:10.1177/13591053251369373)
Supplement: sj-docx-1-hpq-10.1177_13591053251369373 – Supplemental material for The effects of moral distress on burnout and mental well-being across healthcare and care occupations: Do age and work resources matter? [file sj-docx-1-hpq-10.1177_13591053251369373.docx]

# Supplementary Materials for “The Effects of Moral Distress on Burnout and Mental Well-being Across Healthcare and Care Occupations”

## **Table S1.** Evaluation of the formative moral distress construct, after removing item 8

| **Indicator** | **Outer Weight** | ***p*** | **Outer Loading** | ***p*** | **VIF** |
| --- | --- | --- | --- | --- | --- |
| MD1 → Moral Distress | -0.06 | 0.430 | 0.762 | **0.000** | 3.584 |
| MD2 → Moral Distress | 0.251 | **0.002** | 0.834 | **0.000** | 4.162 |
| MD3 → Moral Distress | 0.058 | 0.437 | 0.786 | **0.000** | 3.340 |
| MD4 → Moral Distress | 0.043 | 0.537 | 0.791 | **0.000** | 3.245 |
| MD5 → Moral Distress | 0.243 | **0.001** | 0.885 | **0.000** | 3.465 |
| MD6 → Moral Distress | 0.334 | **0.000** | 0.922 | **0.000** | 3.901 |
| MD7 → Moral Distress | 0.288 | **0.000** | 0.811 | **0.000** | 1.885 |

*Note*. Bold values indicate significant outer weights and outer loadings. *p < .*05.

## **Table S2.** Evaluation of the reflective constructs of exhaustion, disengagement, mental well-being, control, and collegial support

| **Construct** | **Indicator** | **Outer Loading** | **Cronbach’s *α*** | ***ρ_a_*** | ***ρ_c_*** | **AVE** |
| --- | --- | --- | --- | --- | --- | --- |
| **Exhaustion** | Ex2 | 0.717 | 0.855 | 0.859 | 0.888 | 0.502 |
|  | Ex4 | 0.738 |  |  |  |  |
|  | Ex5 | 0.749 |  |  |  |  |
|  | Ex8 | 0.763 |  |  |  |  |
|  | Ex10 | 0.711 |  |  |  |  |
|  | Ex12 | 0.814 |  |  |  |  |
|  | Ex14 | 0.578 |  |  |  |  |
|  | Ex16 | 0.558 |  |  |  |  |
| **Disengagement** | Dis1 | 0.654 | 0.806 | 0.819 | 0.861 | 0.510 |
|  | Dis3 | 0.760 |  |  |  |  |
|  | Dis6 | 0.741 |  |  |  |  |
|  | Dis9 | 0.815 |  |  |  |  |
|  | Dis11 | 0.665 |  |  |  |  |
|  | Dis15 | 0.632 |  |  |  |  |
| **Mental Well-being** | MWB1 | 0.739 | 0.842 | 0.847 | 0.880 | 0.513 |
|  | MWB2 | 0.695 |  |  |  |  |
|  | MWB3 | 0.758 |  |  |  |  |
|  | MWB4 | 0.718 |  |  |  |  |
|  | MWB5 | 0.737 |  |  |  |  |
|  | MWB6 | 0.672 |  |  |  |  |
|  | MWB7 | 0.692 |  |  |  |  |
| **Control** | Co1 | 0.807 | 0.742 | 0.765 | 0.836 | 0.561 |
|  | Co2 | 0.693 |  |  |  |  |
|  | Co3 | 0.773 |  |  |  |  |
|  | Co4 | 0.719 |  |  |  |  |
| **Collegial Support** | SC1 | 0.928 | 0.820 | 0.826 | 0.917 | 0.848 |
|  | SC2 | 0.913 |  |  |  |  |

*Note*. AVE = Average Variance Extracted. *ρ_a_* = Construct reliability (Dijkstra & Henseler's rho). *ρ_c_* = Composite reliability. Outer loadings ≥ 0.7 are considered reliable, while loadings between 0.5 and 0.7 may be retained based on theoretical justification (Hair et al. 2022).

## **Table S3.** Heterotrait-Monotrait (HTMT) ratios of correlations

| **Construct** | **1** | **2** | **3** | **4** | **5** |
| --- | --- | --- | --- | --- | --- |
| **1. Disengagement** | — |  |  |  |  |
| **2. Exhaustion** | 0.709 | — |  |  |  |
| **3. Mental Well-being** | 0.689 | 0.754 | — |  |  |
| **4. Control** | 0.541 | 0.564 | 0.441 | — |  |
| **5. Collegial Support** | 0.440 | 0.413 | 0.437 | 0.358 | — |

***Note***: Values represent the Heterotrait-Monotrait (HTMT) ratio of correlations. Discriminant validity is supported when HTMT values remain below 0.85 for a conservative threshold or 0.90 for a more lenient criterion (Hair et al., 2021).

## **Table S4.** Standardized Path Coefficients across age Groups

|  | ***β* under 30** | ***β* 30-39** | ***β* 40-49** | ***β* 50-59** | ***β* over 60** |
| --- | --- | --- | --- | --- | --- |
| **MoralDistress->Exhaustion** | 0,417 | 0,358 | 0,28 | 0,361 | 0,624 |
| **MoralDistress->Disengagement** | 0,274 | 0,311 | 0,402 | 0,363 | 0,576 |

*Note.* *β****=*** Standardized beta coefficients.

## **Table S5. Age group** differences in standardized path coefficients

|  | ***Δβ* Under 30 - 30-39** | ***p*** | ***Δβ* Under 30 - 40-49** | ***p*** | ***Δβ* Under 30 - 50-59** | ***p*** | ***Δβ* Under 30 - 60 and over** | ***p*** | ***Δβ* 30-39 - 40-49** | ***p*** | ***Δβ* 30-39 - 50-59** | ***p*** | ***Δβ* 30-39 - 60 and over** | ***p*** | ***Δβ* 40-49 - 50-59** | ***p*** | ***Δβ* 40-49 - 60 and over** | ***p*** | ***Δβ* 50-59 - 60 and over** | ***p*** |
| --- | --- | --- | --- | --- | --- | --- | --- | --- | --- | --- | --- | --- | --- | --- | --- | --- | --- | --- | --- | --- |
| **MoralDistress-> Disengagement** | -0,037 | **< .001** | -0,128 | **< .001** | -0,088 | **< .001** | -0,302 | **< .001** | -0,091 | 0,339 | -0,051 | 0,608 | -0,265 | **< .001** | 0,04 | 0,651 | -0,174 | **< .001** | -0,214 | **< .001** |
| **MoralDistress-> Exhaustion** | 0,059 | **< .001** | 0,137 | **< .001** | 0,055 | **< .001** | -0,208 | **< .001** | 0,078 | 0,374 | -0,003 | 0,981 | -0,266 | **< .001** | -0,081 | 0,366 | -0,344 | **< .001** | -0,263 | **< .001** |

*Note.* *Δβ* indicates the difference in standardized path coefficients between age groups. *p*-values reflect significant differences in the paths. Bold values indicate significant differences at *p < .*05.

|  | ***Δf²* Under 30 - 30-39** | ***p*** | ***Δf²* Under 30 - 40-49** | ***p*** | ***Δf²* Under 30 - 50-59** | ***p*** | ***Δf²* Under 30 - 60 and over** | ***p*** | ***Δf²* 30-39 - 40-49** | ***p*** | ***Δf²* 30-39 - 50-59** | | ***p*** | | ***Δf²* 30-39 - 60 and over** | | ***p*** | | ***Δf²* 40-49 - 50-59** | | ***p*** | | ***Δf²* 40-49 - 60 and over** | | ***p*** | | ***Δf²* 50-59 - 60 and over** | | ***p*** |
| --- | --- | --- | --- | --- | --- | --- | --- | --- | --- | --- | --- | --- | --- | --- | --- | --- | --- | --- | --- | --- | --- | --- | --- | --- | --- | --- | --- | --- | --- |
| **MoralDistress -> Disengagement** | -0,001 | **< .001** | -0,09 | **< .001** | -0,041 | **< .001** | -0,365 | **< .001** | -0,089 | 0,381 | -0,04 | | 0,655 | | -0,363 | | **< .001** | | 0,049 | | 0,63 | | -0,275 | | **< .001** | | -0,324 | | **< .001** |
| **MoralDistress -> Exhaustion** | 0,147 | **< .001** | 0,191 | **< .001** | 0,138 | **< .001** | -0,255 | **< .001** | 0,044 | 0,598 | -0,008 | | 0,922 | | -0,402 | | **< .001** | | -0,052 | | 0,515 | | -0,446 | | **< .001** | | -0,394 | | **< .001** |
| *Note.* *Δf²* indicates the difference in effect between age groups. p-values reflect significant differences in the paths. Bold values indicate significant differences at p < .05. | | | | | | | | | | | |  | |  | |  | |  | |  | |  | |  | |  | |  |  |

## **Table S6. Age group** differences in effect sizes
